# Differential Effect of Sucrose and Fructose in Combination with a High Fat Diet on Intestinal Microbiota and Kidney Oxidative Stress

**DOI:** 10.3390/nu9040393

**Published:** 2017-04-16

**Authors:** Adriana Rosas-Villegas, Mónica Sánchez-Tapia, Azalia Avila-Nava, Victoria Ramírez, Armando R. Tovar, Nimbe Torres

**Affiliations:** 1 Departamento de Fisiología de la Nutrición, Instituto Nacional de Ciencias Médicas y Nutrición Salvador Zubirán, México D.F. 14080, Mexico; kadriana.rosas@gmail.com (A.R.-V.); qfbmoniktc@gmail.com (M.S.-T.); zomi33@gmail.com (A.A.-N.); tovar.ar@gmail.com (A.R.T.); 2 Departamento de Nefrologia, Instituto Nacional de Ciencias Médicas y Nutrición Salvador Zubirán, México D.F. 14080, Mexico; vikka60@hotmail.com

**Keywords:** microbiota, renal oxidative stress, sucrose, fructose, obesity, LPS, inflammation

## Abstract

There is controversial information about the adverse effect of sucrose (S) or fructose (F) in the development of obesity. Thus, the purpose of the study was to evaluate the effect of S or F in a high fat diet (HF) on gut microbiota and renal oxidative stress. Rats were fed for four months with either high-fat + sucrose (HFS) or high-fat + fructose (HFF) or a control diet (C). Half of the HFS or HFF groups were maintained with the same diet and the other half were switched to the consumption of C. HFS and HFF groups increased 51% and 19% body weight, respectively, compared with the C group. Body fat mass, metabolic inflexibility, glucose intolerance, lipopolysaccharide (LPS), insulin, renal reactive oxygen species (ROS), malondialdehyde (MDA), *Nadphox*, and *Srebp-1* were significantly higher and antioxidant enzymes and lean body mass were significantly lower in the HFS group with respect to the HF-F group. Change in the consumption of HFS or HFF to a C diet ameliorated the insulin and glucose intolerance. The type of carbohydrate differentially modified the microbiota composition, however, both groups significantly decreased *C. eutactus* with respect to the C group. Thus, metabolic alterations with the HFS diet had a more detrimental effect than HFF.

## 1. Introduction

Since 1980, overweight and obesity have more than doubled in the worldwide population [1]. In addition to the decrease of physical activity in a genetically-stable population, increased consumption of foods with a high content of energy, mainly attributed by sugar and fat, constitutes the main environmental factor, contributing to this dramatic increase in obesity that has reached epidemic levels [2]. Obesity is often accompanied by hyperinsulinemia and, consequently, dyslipidemia, which could impact the renal structures, like the glomerulus, contributing to vasodilation and hypertension, leading to kidney injury [3]. 

In recent years, the modification of gut microbiota and activation of inflammation pathways have been implicated in the development of insulin resistance and renal disease related to obesity [4]. Kidney disease is associated with low fiber consumption and increased intestinal permeability due to a modified intestinal microbiota [5]. During obesity there is an inflammatory state and imbalance in the gut microbiota composition (dysbiosis) involving the toll-like receptor (TLR) family. Obesity results in an increase of circulating lipopolysaccharide (LPS) because of the disruption of the enterocyte junctions resulting in increased gut permeability [4]. Another mechanism involved in the kidney injury is the protein fermentation of certain bacteria that result in an increase in uremic toxins, such indoxyl sulfate and *p*-Cresyl sulfate [6]. TLR-4 is an important component of the innate immune system signaling through different ligands that recognize, among others, LPS, associated with kidney disease [7,8]. Saturated fatty acids [8], fructose [9,10], or sucrose can activate inflammatory pathways observed in obesity and kidney injury [7,8]. LPS, in turn, induces insulin resistance through the activation of the transcription factor NF-κB (nuclear factor kappa-beta) which promotes the expression of inflammatory cytokines [4], such as tumor necrosis factor-α (TNF-α), interleukin-1β (IL-1β), and interleukin-6 (IL-6), which contribute to the impairment of insulin signaling, affecting kidney function [11]. The production of these cytokines leads to a redox imbalance and an increase in reactive oxygen species (ROS), establishing a continuous cycle of inflammation and oxidative stress [12,13,14] implicated in chronic kidney disease (CKD). Interestingly, a recent study suggests that obesity and kidney damage are associated with insulin-resistant obese individuals. During insulin resistance there is an increased production of radicals and a decrease of some antioxidant enzymes [12,15]. ROS impairs insulin signaling by inhibiting GLUT 4 translocation to the cell membrane [16,17]. The increase in ROS concentration induces lipid peroxidation that contributes to oxidative stress with malondialdehyde (MDA) and the accumulation of ROS in the blood as end products [13,14]. There is sufficient information about the effect of sucrose or fructose on obesity [18] and its consequences on inflammation and oxidative stress [19]. However, part of the increase in obesity, worldwide, is caused by the consumption of a high fat diet and sugars [2], yet there is less information available about the differential effects of sugars in combination with a high fat diet on gut microbiota and kidney damage [20]. 

It is still not understood whether the consumption of a high fat diet and different kinds of sugars can cause differential effects on diet-induced obesity. Thus, the purpose of the present study was to assess the long-term effects (four months) of a high fat diet in combination with ad libitum access to one of two sweetened solutions (5% sucrose or 5% fructose) on the onset of obesity and metabolic abnormalities that impact the intestinal microbiota and the kidney oxidative stress on Wistar rats. 

## 2. Materials and Methods

### 2.1. Animals 

Male Wistar rats aged 5–7 weeks were obtained from the National Institute of Medical Sciences and Nutrition. The animals were housed in individual cages and maintained at a controlled room temperature with 12-h light-dark cycles and free access to water and food. The study was developed in two stages. In the first stage rats were randomized into three groups; eight rats were fed a high-fat diet and 5% sucrose in drinking water (HFS), eight rats were fed a high-fat diet and 5% fructose in drinking water, and eight rats were fed a control diet (C) [21] for four months. In the second stage, four rats fed with HFS or four rats HFF were changed to the control diet, (HFS-C) and (HFF-C), respectively (*n* = 4 per group) for two months. The control group continued consuming the control diet (*n* = 8). Animal weight and food consumption were determined every other day during the protocol. At the end of the study urine and stool of each rat were collected and stored at −70 °C until analysis. At the end of the experiment, the rats were killed by decapitation after being anesthetized with CO_2_. The kidney was rapidly removed and stored at −70 °C until analysis. Serum was obtained by centrifugation of blood at 1500× *g* for 10 min and stored at −70 °C until further analysis. The Animal Committee of the National Institute of Medical Sciences and Nutrition, Mexico City (CINVA1444) approved the procedure.

### 2.2. Gut Microbiota Profiling

Fresh feces samples were collected immediately, frozen, and stored at −70 °C until use. Bacterial DNA content was extracted using the QIAamp DNA Mini Kit (Qiagen, Valencia, CA, USA) according to the manufacturer’s instructions. 

Bacterial DNA was amplified by PCR with barcode universal bacterial primers targeting variable regions 3–4 of the 16S rRNA gene. We used the specific forward and reverse primers: 5’-TCGTCGGCAGCGTCAGATGTGTATAAGAGACAGCCTACGGGNGGCWGCAG-3’, 5’-GTCTCGTGGGCTCGGAGATGTGTATAAGAGACAGGACTACGGGTATCTAATCC-3’. Samples were pooled and sequenced with the Illumina MiSeq platform (MiSeq Reagent Kit V.3, 600 cycles, San Diego, CA, USA) according to the protocol suggested by Illumina (16S metagenomic sequencing library preparation).

#### Sequence processing

For taxonomic composition analysis, custom C# and Python scripts, as well as Python scripts in the Quantitative Insights Into Microbial Ecology (QIIME, San Diego, CA, USA) software pipeline 1.9, were used to process the sequencing files. The sequence outputs were filtered for low-quality sequences (defined as any sequences that are <200 bps or >600 bps, sequences with any nucleotide mismatches to either the barcode or the primer, sequences with homopolymer runs >6, sequences with an average quality score of <25, and sequences with ambiguous bases >0) and were truncated at the reverse primer. Sequences were chimera checked with Chimera Slayer, and chimeric sequences were filtered out. Analysis started by clustering sequences within a percent sequence similarity into operational taxonomic units (OTUs) with a 97% similarity threshold. Thus, 99.68%, 98.1%, 98.08%, 82.74%, 56.43%, and 14.82% of the reads were assigned to the phylum, class, order, family, genus, and species levels, respectively. Species richness (Observed, Chao1) and alpha diversity measurements (Shannon) were calculated, and we estimated the within-sample diversity at a rarefaction depth of 5495 reads per sample. Weighted and unweighted UniFrac distances were used to perform the principal coordinate analysis (PCoA). Differences in the relative abundance at the phylum, family, genus, and species levels were compared using a Student’s *t*-test for independent samples.

### 2.3. Biochemical Parameters 

Serum biochemical parameters including glucose, triglycerides, total, and LDL cholesterol were analyzed with a COBAS C11 (Roche, Basel, Switzerland). Serum insulin (Alpco Diagnostics, Salem, NH, USA) and LPS (Cloud-Clone Corp, Houston, TX, USA) were determined using commercial ELISA kits.

### 2.4. Glucose Tolerance Test

The glucose tolerance test was determined as previously reported [22] by the administration of an intraperitoneal injection of a glucose load of 2 g per kg body weight in fasted rats. The blood samples were collected from the tail vein at 0, 15, 30, 45, 60, 90, and 120 min after administration of the glucose. Plasma glucose concentration was measured using a OneTouch Ultra Glucose Meter (LifeScan, Inc., Milpitas, CA, USA) 

### 2.5. Energy Expenditure 

Energy expenditure was determined by indirect calorimetry in an Oxymax Lab Animal Monitoring (CLAMS) System (Columbus Instruments, Columbus, OH, USA). The animals were individually housed in plexiglass cages with an open-flow system connected to the CLAMS. Throughout the test, O_2_ consumption (VO_2_ mL/kg/h) and CO_2_ production (VCO_2_, mL/kg/h) were measured sequentially for 90 s. The respiratory exchange ratio (RER) was calculated as the average ratio of VCO_2_ produced to VO_2_ inhaled (VCO_2_/VO_2_).

### 2.6. Western Blot Analysis

Total protein of pooled kidney samples (*n* = 4) was extracted and quantified by Bradford assay (Bio-Rad, Hercules, CA, USA) and stored at −70 °C. The protein detection was performed by electrophoresis in SDS-PAGE and then transferred to polyvinylidene difluoride membranes. All blots were blocked with 5% nonfat dry milk (Bio-Rad, Hercules, CA, USA) for 60 min at room temperature and incubated overnight at 4 °C with the following antibodies: toll-like receptor 4 (TLR-4) (1:10,000), nuclear factor-kappa B (NF-κB) (1:3000), and tumor necrosis factor α/β (TNF-α) (1:2000). The blots were incubated with anti-rabbit, anti-goat, or anti-mouse secondary antibodies conjugated with horseradish peroxidase (1:3500). Actin (1:5000) was used to normalize the data. Images were analyzed with a ChemiDocTM XRS + System Image LabTM Software (Bio-Rad, Hercules, CA, USA). The assays were performed three times using independent blots.

### 2.7. Renal Gene Expression 

The gene expression was determined by real-time PCR. First, total RNA was extracted using TRIzol, following the manufacturer’s instructions. The mRNA abundance was measured by real-time quantitative PCR using Taqman or SYBR^®^ Green assays (Applied Biosystems, Foster, CA, USA), using *HPRT* and *cyclophilin* as references for normalization (Table 1).

### 2.8. Oxidative Markers

Reactive oxygen species (ROS) in kidney were measured by a fluorescence method. A 50 μL homogenate was incubated with 150 μL of fluorescent compound 5-(and-6) carboxy-2,7-dichlorofluorescein (carboxy-DCF) (5 μM) for 1 h at 37 °C. Fluorescence was measured in a Synergy HT multimode microplate reader (Biotek, Winooski, VA, USA). The data were expressed as fluorescence units/mg of protein [23]. MDA concentration was measured spectrophotometrically. A solution of 1-methyl-2-phenylindole was diluted in acetonitrile: methanol (3:1) was added to 300 μL of kidney homogenate, then 150 μL of HCl (37%) were added and incubated for 40 min at 45 °C and the optical density was measured at 586 nm. Data were expressed as nmol MDA/mg protein [24]. The hydrogen peroxide in urine was measured by using Amplex red. The assay was performed with 25 μL of urine and 50 μL of the reaction mixture (0.1 mM Amplex Red, HRP 0.2 U mL^−1^) and incubated for 30 min in the dark at room temperature. Finally, the fluorescence intensity was measured in a Synergy HT multimode microplate reader (Biotek, Winooski, VA, USA). The results were expressed by μM/mL of urine.

### 2.9. Statistical Analysis

The results were expressed as the mean ± SEM. Statistical analysis was performed using one-way ANOVA followed by Bonferroni’s post-hoc test, using Prism 5.0 software (GraphPad, San Diego, CA, USA); *p* < 0.05 was considered significant.

## 3. Results

### 3.1. Body Composition and Energy Expenditure

**Figure 1:**
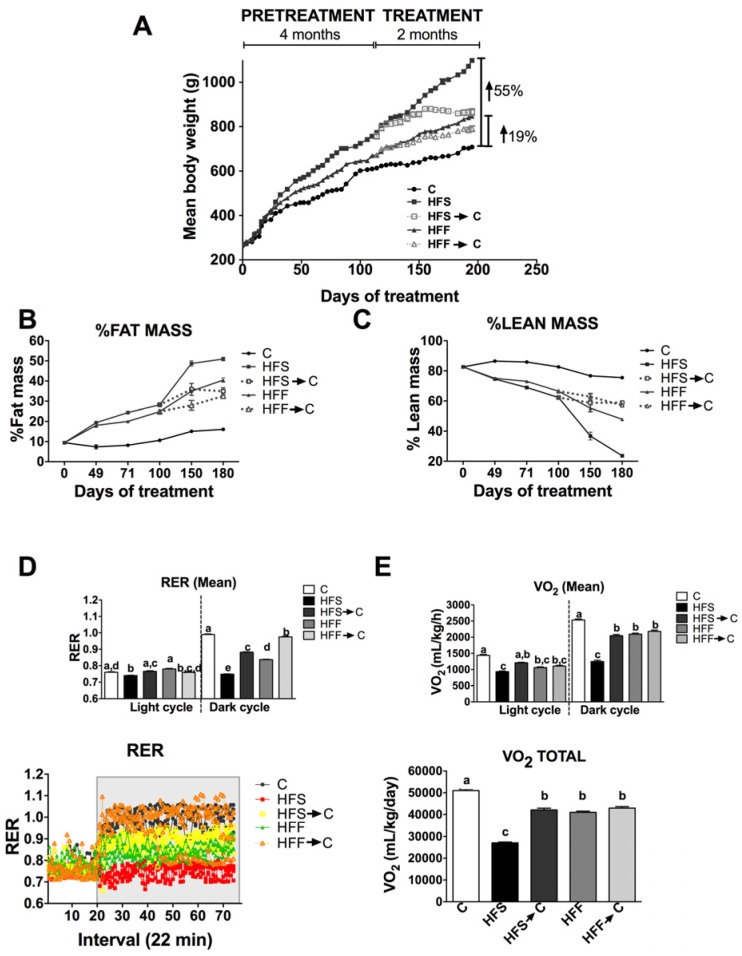
Effect of a high fat diet enriched with 5% of sucrose (HFS) or fructose (HFF) in water on body weight (**A**); fat mass (**B**); lean mass (**C**); energy expenditure (**D**); and oxygen consumption (**E**). The data are expressed as the mean ± SEM (*n* = 4). Results were considered statistically significant at *p* < 0.05. The differences between groups are indicated by letters, where a > b > c. C: control; HFS: high-fat and sucrose; HFS→C high-fat and sucrose, switched to the control diet; HFF: high-fat and fructose; HFS→C: high-fat and fructose, switched to control diet.

After six months, the HFS and HFF groups showed 55% and 19% weight gain with respect to the C group (Figure 1A). When the HFS and HFF groups were switched for two months to the control diet, there was a significant decrease in body weight by 22% and 7%, respectively. Body weight gain was related with changes in the body composition. Remarkably, 51% of the body in the HFS group was fat mass and only 24% was lean mass, whereas the HFF group had 40% fat mass and 48% lean mass, indicating that the presence of sucrose in the diet produced a more deleterious effect than the fructose in the development of fat mass (Figure 1B,C). These adverse effects were, in part, reverted by the consumption of an adequate diet for rodents (Figure 1B,C). These alterations in body composition were accompanied by metabolic alterations. The HFS group showed marked metabolic inflexibility, i.e., the inability to switch substrates during the feeding period, with lipids being the main substrate, whereas the HFF group showed a less severe metabolic inflexibility compared with C group (Figure 1D, *p* < 0.05). These changes were associated with lower oxygen consumption (Figure 1E).

### 3.2. Analysis of Microbiota Composition

**Figure 2:**
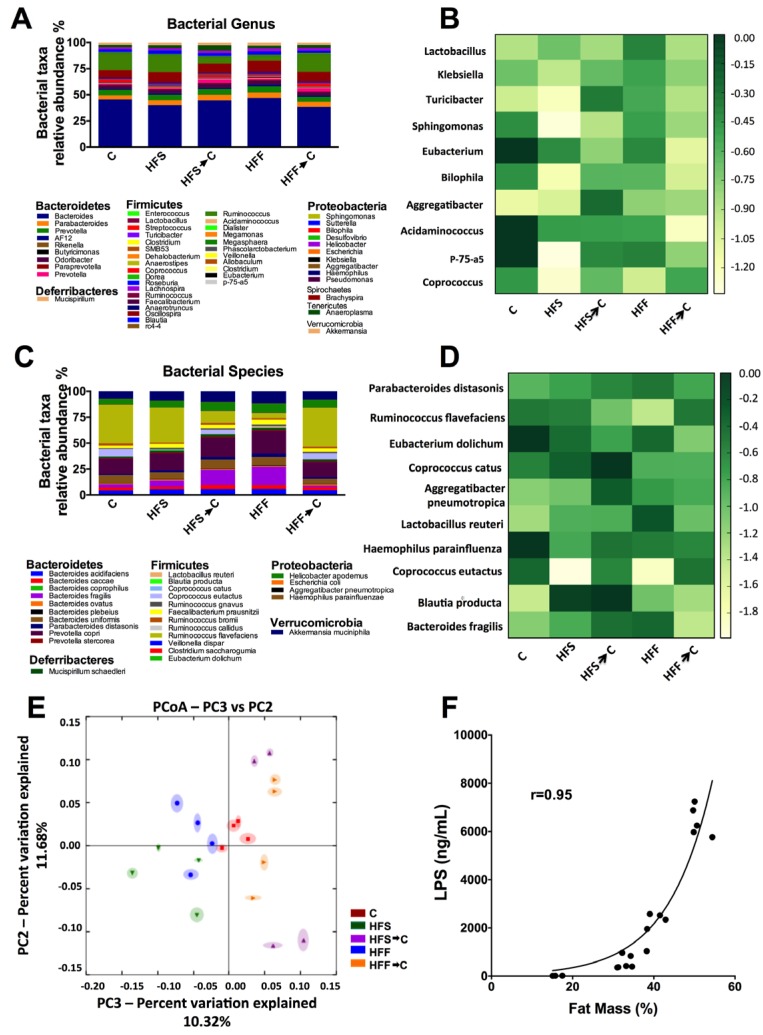
High-fat sucrose (HFS) or high-fat fructose (HFF) diets differentially modified the gut microbiota. Relative abundances of the gut microbiota at the bacterial genus (**A**); heat map showing the 10 most modified genera (**B**); relative abundances at the specie level (**C**); and heat map showing the ten most modified species (**D**) after the consumption of HFS or HFF diets. Unweighted principal component analysis (PCA) of gut microbiota after the consumption of different diets (**E**). The closer the spatial distance between samples the more similar they are with respect to both axes (PERMANOVA, *p* = 0.001). Correlation between serum LPS concentration and percent fat mass (*r* = 0.95) (**F**). The data are expressed as the mean ± SEM (*n* = 4). Results were considered statistically significant at *p* < 0.05. The differences between groups are indicated by letters, where a > b > c. C: control; HFS: high-fat and sucrose; HFS→C: high-fat and sucrose, switched to the control diet; HFF: high-fat and fructose; HFS→C: high-fat and fructose, switched to control diet.

There is a controversy about the adverse effect of different types of simple carbohydrates in a high fat diet on the development of obesity mediated by changes in gut microbiota. The results of the present work showed that the main phyla in the different groups studied were Bacteroidetes, Firmicutes, and Proteobacteria. The main bacterial genus modified by the consumption of HFS or HFF were *Coprococcus*, *Acidaminococcus*, and *Eubacterium*. Particularly, the HFS group showed a marked decreased in P75-a5, *Aggregatibacter*, *Bilophila*, *Sphingomonas*, *Turicibacter*, and *Klebsiella* with respect to the C group. The HFF groups showed a less pronounced effect on these genera. At the species level, a heat map was created based on the ten most modified species. There was a remarkable decrease in *C. eutactus* in the HFS and HFF groups, and there was a significant increase in *L. reuteri* and *B. fragilis* in HFF group. Particularly, *B. producta* was increased in the HFS group and *R. flavefasciens* in the HFF group (Figure 2A–D). Clustering the bacterial communities using principal component analysis (PCA) revealed that the microbiota after the consumption of HFS or HFF diets was different to that of the C group (ANOSIM *R* = 0.56, *p* = 0.001) (Figure 2E). However, when the HFS or HFF groups switched to the control diet, the gut microbiota, in part, returned to a similar extent to that of the C group. The amount of body fat was associated (*r* = 0.95) with the concentration of serum LPS (Figure 2F).

### 3.3. Biochemical Parameters and Glucose Tolerance 

**Figure 3:**
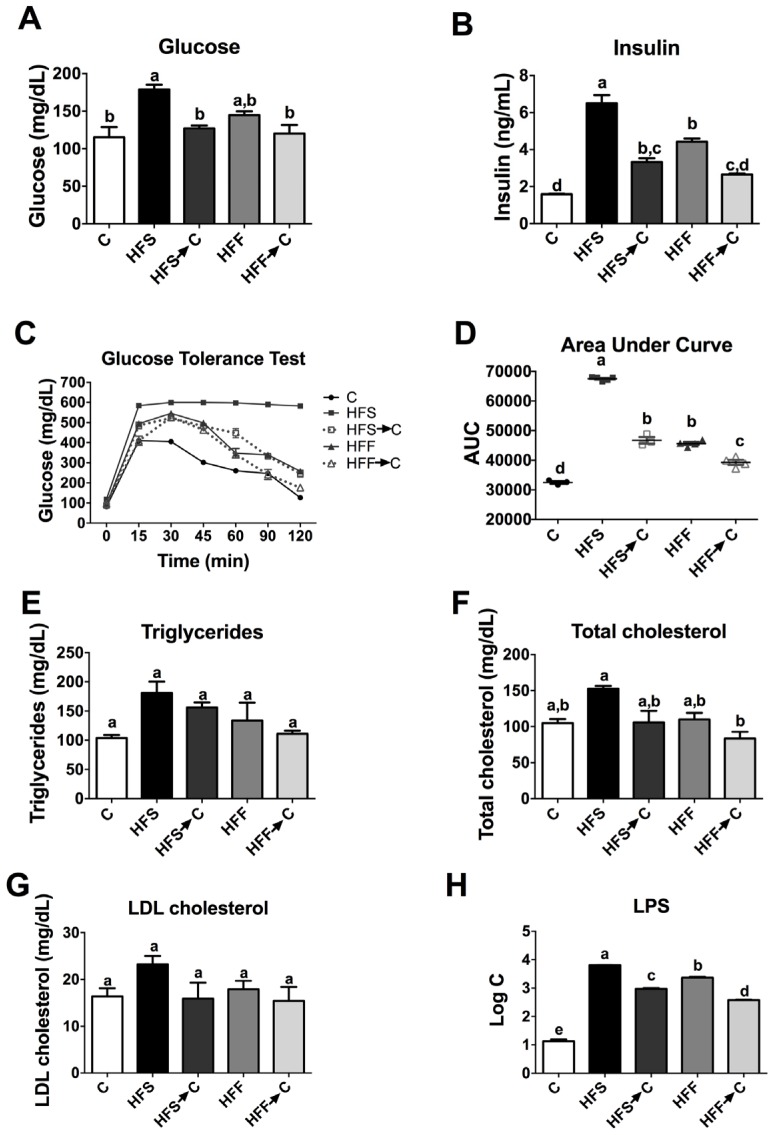
Serum biochemical parameters and glucose tolerance test after the consumption of different diets. Serum glucose (**A**); insulin (**B**); glucose tolerance test (**C**); area under the curve after the glucose tolerance test (**D**); triglycerides (**E**); total cholesterol (**F**); LDL cholesterol (**G**); and lipopolysaccharides (**H**). The data are expressed as the mean ± SEM (*n* = 4). Results were considered statistically significant at *p* < 0.05. The differences between groups are indicated by letters, where a > b > c. C: control; HFS: high-fat and sucrose; HFS→C: high-fat and sucrose, switched to control diet; HFF: high-fat and fructose; HFS→C: high-fat and fructose, switched to control diet.

The consumption of a high-fat diet enriched with sugars could produce biochemical alterations to different extents. We observed that the HFS group showed the highest levels of serum glucose, insulin, triglycerides, total cholesterol, and LDL cholesterol with respect to the C group (*p* < 0.05). Interestingly, the HFF group showed the same trend as the HFS group, but to a lesser extent (Figure 3). Only the HFS group showed glucose intolerance with the highest value of the area under the curve (AUC) after the glucose tolerance test. Remarkably, the HFS group showed extremely high levels of LPS (6310 ng/mL), 489-fold higher than the C group, followed by the HFF group, 192-fold higher than the C group, indicating a severe metabolic endotoxemia.

### 3.4. Inflammation Markers and Oxidative Stress 

**Figure 4:**
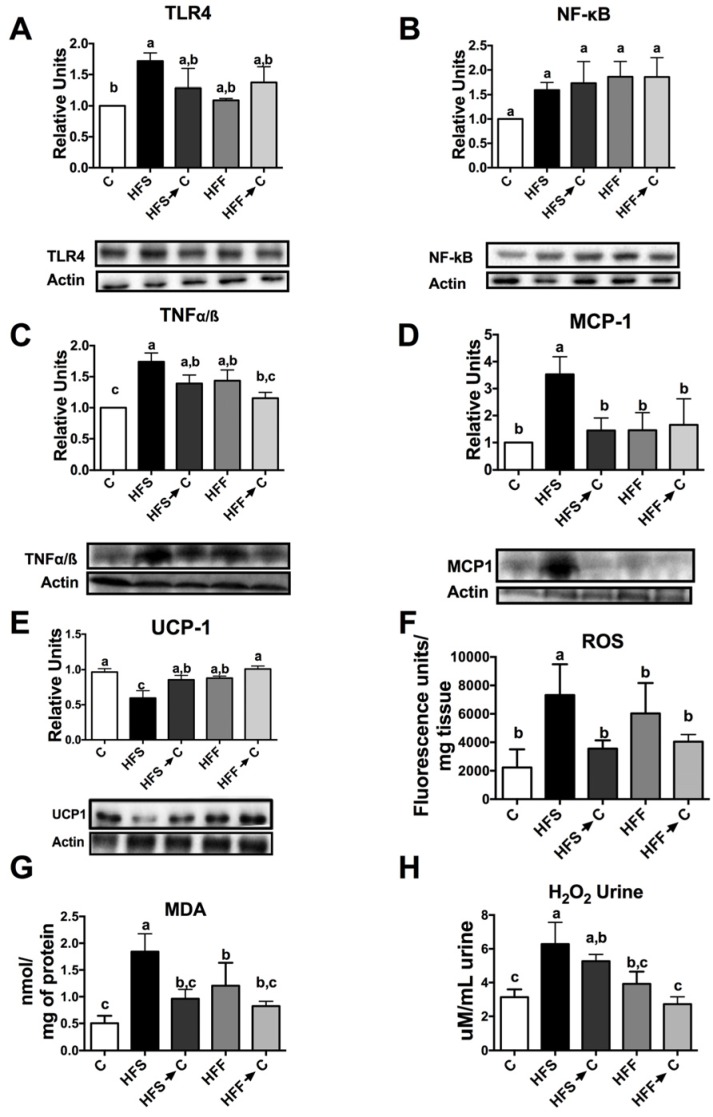
Relative protein abundance and oxidative markers in the kidneys of rats fed high-fat sucrose or fructose diets. Protein abundance of toll-like receptor 4 (**A**); nuclear factor kappa B (**B**); tumor necrosis factor alpha (**C**); monocyte chemoattractant protein 1 (**D**); and uncoupling protein 1 (**E**). Renal reactive oxygen species (**F**); malondialdehyde concentration (**G**); and urinary hydrogen peroxide (**H**). The data are expressed as the mean ± SEM (*n* = 4). Results were considered statistically significant at *p* < 0.05. The differences between groups are indicated by letters, where a > b > c. C: control; HFS: high-fat and sucrose; HFS→C: high-fat and sucrose, switched to the control diet; HFF: high-fat and fructose; HFS→C: high-fat and fructose, switched to the control diet.

The continuous exposure to a high-fat–high-sugar diet produce metabolic alterations in the liver, adipose tissue, and skeletal muscle, however, kidneys could also be affected by this chronic inflammatory state. As expected, the HFS group increased the inflammatory markers TLR-4, NF-κB, TNFα/β, and MCP1 compared with the C group, followed by the HFF group (Figure 4A–D, *p* < 0.05), on the contrary, we found a significant decrease in kidney UCP-1 only in the HFS group (Figure 4E). In addition, the consumption of HFS or HFF diets significantly increased ROS levels and MDA concentration in the kidney compared to the C group (Figure 4F,G). However, this effect was reverted by the consumption of the C diet. Nevertheless, the HFS group showed more oxidative damage, which was reflected in the levels of urine H_2_O_2_ excreted in comparison with the HFF group (Figure 4H).

### 3.5. Antioxidant System

**Figure 5:**
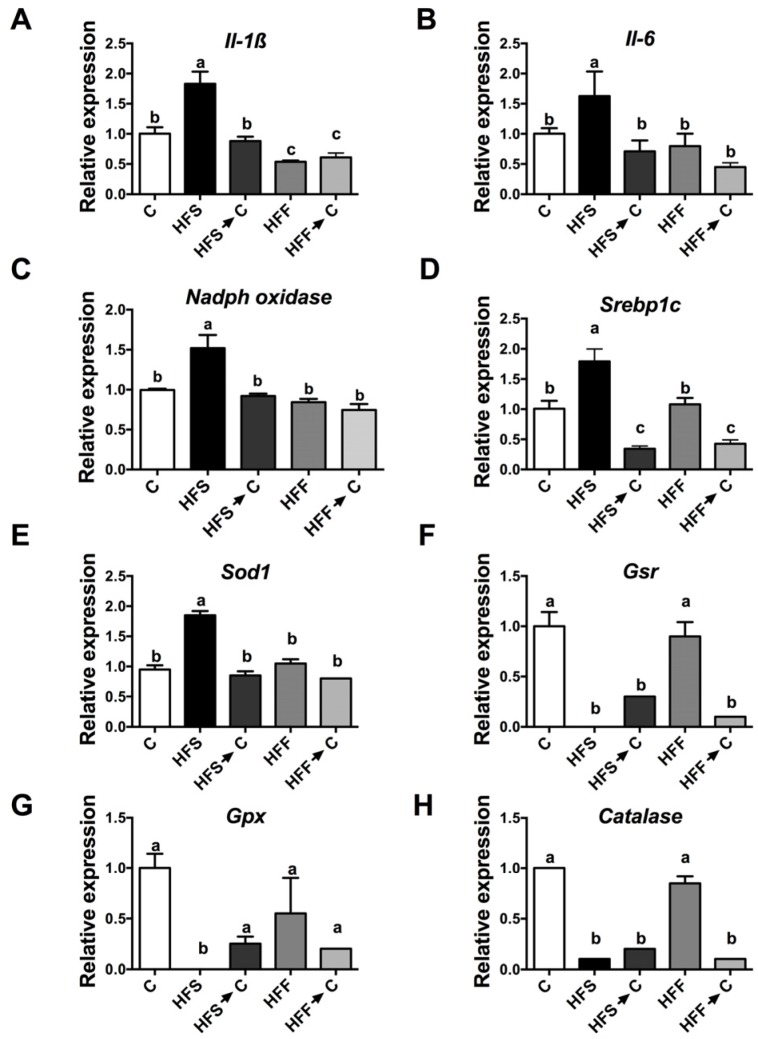
Relative gene expression of inflammatory cytokines, lipogenesis, oxidant, and antioxidant enzymes in kidney. Interleukin 1-β (**A**); interleukin 6 (**B**); NADPH oxidase (**C**); sterol regulatory element-binding protein-1c (**D**); superoxide dismutase 1 (**E**); glutathione reductase (**F**); glutathione peroxidase (**G**); and catalase (**H**). The data are expressed as the mean ± SEM (*n* = 5). Results were considered statistically significant at *p* < 0.05. The differences between groups are indicated by letters, where a > b > c. C: control; HFS: high-fat and sucrose; HFS→C: high-fat and sucrose, switched to the control diet; HFF: high-fat and fructose; HFS→C: high-fat and fructose, switched to the control diet.

Oxidative stress may be produced by an imbalance between ROS and the antioxidant system, generating inflammation. This, in turn, can produce an elevation of inflammatory cytokines. The HFS group showed the highest increase in IL-1β and IL-6 with respect to the C group (Figure 5A,B, *p* < 0.05). These results were associated with an increase in renal gene expression of NADPH oxidase, considered as a pro-oxidant enzyme (Figure 5C). On the other hand, it has been proposed that the increase in renal lipogenesis could be responsible for the inflammation. The HFS group showed the highest expression of renal Srebp1c, a transcription factor involved in lipogenesis (Figure 5D). During this inflammation process, there was an imbalance between ROS and antioxidant enzymes, such as catalase, glutathione peroxidase (Gpx), glutathione reductase (Gr), and superoxide dismutase 1 (Sod1). The HFS group showed a significant reduction in the expression of Cat, Gpx, and Gr (Figure 5E–H), while the HFF group maintained similar levels of these enzymes as the control group, suggesting a lesser oxidative stress than the HFS group.

## 4. Discussion

Obesity is a serious health problem in the world and is associated with a significant increase of all-cause mortality [25] accompanied by inflammation and frequently by disease. One of the main causes of obesity, besides physical inactivity, is the consumption of foods high in saturated fat and simple carbohydrates. Additionally, there is a debate on whether the specific type of carbohydrate consumed is responsible for the development of obesity. Increased intake of certain macronutrients, such as fat and simple carbohydrates, and particularly fructose, have been claimed to be risk factors for the development of kidney disease, hypertension, and obesity among others, due to the high consumption of high fructose corn syrup (HFCS) used in beverages [26]. However, the composition of the high fructose corn syrup is 42% fructose and 53% glucose [27], similar to the composition of sucrose. Sucrose and HFCS deliver fructose and glucose in similar ratios to the same tissues. During digestion, sucrose is hydrolyzed to free glucose and fructose by the enzyme sucrase present in the small intestine. Then, glucose and fructose are transported into the portal circulation through the transporters SGLT-1, GLUT5, and GLUT2 on the enterocytes [28]. Whilst there is rising evidence about the deleterious effect of sucrose in the development of obesity, cardiovascular disease, and diabetes, sucrose intake is not solely responsible for obesity, but the increase in other macronutrients like saturated fat and energy [25]. Results of several animal and human studies suggest that intestinal bacteria overgrowth may be involved in pathologies, including non-alcoholic fatty liver disease, and increases in LPS binding protein that are associated with a marked increase expression of TNF-α [29]. All of this evidence suggested that the combination of a high fat-high sucrose diet is responsible, in part, for the development of obesity. The results of the present work indicated differential effects between the disaccharide sucrose and the monosaccharide fructose in combination with a high fat diet. One of the striking results of the present study was that the consumption of high fat-high sucrose or fructose diets significantly increased fat mass (51% and 40% in rats fed HFS or HFF diet, respectively). The amount of body fat was strongly associated (*r* = 0.95) with the concentration of serum LPS (Figure 2F). 

**Figure 6:**
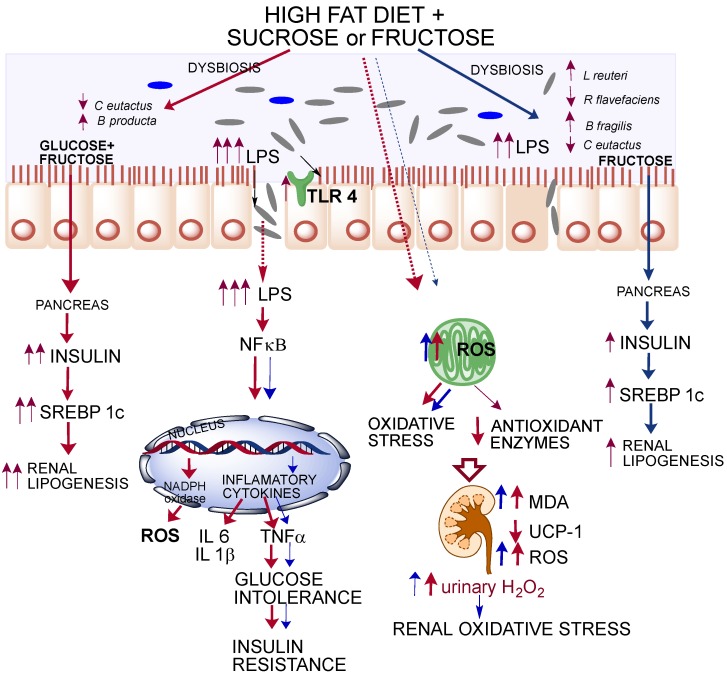
Graphical summary of differential effects of high fat + sucrose of high fat + fructose on gut microbiota, inflammatory cytokines, insulin resistance, and oxidative stress and lipogenesis in kidney.

These results demonstrated that obesity caused by consumption of HFS or HFF diets generated a chronic state of high-grade inflammation mediated by LPS. This state could be originated by dysbiosis in the gut microbiota (Figure 6). Animals fed HFS or HFF diet showed a significant increase in *B. producta* and a significant decrease in *C. eutactus* associated with irritable bowel syndrome [30]. The genus *Blautia* has been associated with phenylacetylglutamine, circulating metabolite derived from bacterial protein fermentation found in early renal function decline [31]. The undesirable effects of the HFF diet was possibly attenuated by the increase in *L. Reuteri* and *B. fragilis* involved in the inhibition of the growth of pathogenic bacteria [32], insulin sensitivity [33], and intestinal epithelium integrity [34]. During the development of obesity, dysbiosis in gut microbiota increased the production of LPS mainly in the HFS group, which could activate TLR4 and promote the induction of NF-κB, provoking the expression of inflammatory cytokines and ROS production. Increases in IL-1β, IL-6, and TNF-α are associated with insulin resistance and glucose intolerance [35]. This inflammatory process increased NADPH oxidase, which increased ROS formation and modified the expression of antioxidant enzymes to a different extent, however, the HFS group showed the most pronounced alteration. These modifications in the antioxidant response were associated with a redox imbalance promoting a vicious cycle in oxidative stress. Imbalance between the ROS levels and antioxidant enzymes led to the formation of MDA, which is a marker of lipid peroxidation. High levels of renal MDA were associated with kidney stress and high production of urinary H_2_O_2_. Interestingly, we found a significant decrease in kidney UCP-1 (Figure 4E). UCP-1 is involved in the leakage of the proton gradient preventing overproduction of mitochondrial ROS. Given that the five UCP family members are identified as able to control ROS [36], when UCP-1 decreases, the ROS control mechanism is impaired. Rats fed a HFS diet showed a significant decrease in renal UCP-1, suggesting an increase in oxidative stress. Finally, another possible mechanisms for which HFS or -F diets increase oxidative stress is by the induction of the transcription factor SREBP-1 involved in renal lipogenesis which, in turn, increases the expression of NADPH oxidase and the production of ROS. The results of the present work indicate that the combination of a high-fat diet and sucrose, and potentially fructose, can produce renal oxidative stress and a severe metabolic endotoxemia produced by a dysbiosis in the gut microbiota. Oxidative stress in the kidney does not reach kidney damage, since KIM-1, a biomarker for renal proximal tubule injury [37], was not modified (data not shown). Importantly, these deleterious effects can be partially reverted or ameliorated by the consumption of an adequate diet. 

## 5. Conclusions

The combination of a high-fat with sucrose (HFS) or fructose (HFF) diet differentially modified the gut microbiota and increased the paracellular transport of LPS generating a chronic state of high-grade inflammation. The HFS diet increased to a higher extent renal lipogenesis and inflammatory markers compared with the HFF diet. As a consequence, there was an increase in glucose intolerance and insulin resistance. Consumption of HFS or HFF diets increased the formation of reactive oxygen species (ROS) and renal oxidative stress.

## Figures and Tables

**Table 1:** Primers used in real-time PCR.

Gene	Primer (5’-3’)	Sequence
*Srebp-1c*	Forward	CGTTGTACTGCAGCCACACT
	Reverse	AGTGGTACTGTGGCCAGGAT
*Nadph oxidase*	Forward	GTCCCTTTGGCACAGTCAGT
	Reverse	AGGCACCCGTCTCTCTACAA
*ucp-1*	Forward	CCGAAACTGTACAGCGGTCT
	Reverse	TGACCTTCACCACCTCTGTG
*tlr-4*	Forward	GTGCCCCGCTTTCAGCTTTG
	Reverse	GTGCCTCCCCAGAGCATTGT
*Il-1beta*	Forward	CAGCAGCATCTCGACAAGAG
	Reverse	CATCATCCCACGAGTCACAG
*Il-6*	Forward	ACCACCCACAACAGACCAGT
	Reverse	CGGAACTCCAGAAGACCAGA
*hprt*	Forward	CTGGTGAAAAGGACCTCTCG
	Reverse	GGCCACATCAACAGGACTCT
*Cyclophilin*	Forward	CGTGGGCTCCGTTGTCTT
	Reverse	TGACTTTAGGTCCCTTCTTCTTATCG
**Fluorogenic probes TaqMan**		
*Catalase (Cat)*	Rn01423343_m1	
*Glutathione peroxidase (Gpx)*	Rn00588153_m1	
*Glutathione reductase (Gr)*	Rn99999088_g1	
*Superoxide dismutase 1 (Sod1)*	Rn00560930-m1	
